# Simultaneous detection and comprehensive analysis of HPV and microbiome status of a cervical liquid-based cytology sample using Nanopore MinION sequencing

**DOI:** 10.1038/s41598-019-55843-y

**Published:** 2019-12-18

**Authors:** Lili Quan, Ruyi Dong, Wenjuan Yang, Lanyou Chen, Jidong Lang, Jia Liu, Yu Song, Shuiqing Ma, Jialiang Yang, Weiwei Wang, Bo Meng, Geng Tian

**Affiliations:** 1 Department of Gynaecology and Obstetrics, Sanmenxia Central Hospital of Henan University of Science and Technology, Sanmenxia, 472000 Henan China; 2 Geneis (Beijing) Co.Ltd, Beijing, 100102 China; 3 Department of Gynaecology and Obstetrics, Peking Union Medical College Hospital, Beijing, 100730 China

**Keywords:** Microbiology techniques, Microbiology techniques, Cervical cancer, Cervical cancer

## Abstract

Human papillomavirus (HPV) is a major pathogen that causes cervical cancer and many other related diseases. HPV infection related cervical microbiome could be an induce factor of cervical cancer. However, it is uncommon to find a single test on the market that can simultaneously provide information on both HPV and the microbiome. Herein, a novel method was developed in this study to simultaneously detect HPV infection and microbiota composition promptly and accurately. It provides a new and simple way to detect vaginal pathogen situation and also provide valuable information for clinical diagnose. This approach combined multiplex PCR, which targeted both HPV16 E6E7 and full-length 16S rRNA, and Nanopore sequencing to generate enough information to understand the vagina condition of patients. One HPV positive liquid-based cytology (LBC) sample was sequenced and analyzed. After comparing with Illumina sequencing, the results from Nanopore showed a similar microbiome composition. An instant sequencing evaluation showed that 15 min sequencing is enough to identify the top 10 most abundant bacteria. Moreover, two HPV integration sites were identified and verified by Sanger sequencing. This approach has many potential applications in pathogen detection and can potentially aid in providing a more rapid clinical diagnosis.

## Introduction

More and more diseases have been shown to be associated with certain kinds of pathogens, including viruses and microbiomes^1,2^. Microbes can contribute to digestive disorders (e.g., inflammatory bowel disease) and disease processes, such as an association with the degenerative lesions seen in Parkinson’s disease, autism, depression^3^ and carcinomagenesis^4^. Furthermore, in gastric cancer, the presence of *Helicobacter pylori* is considered a major contributing factor^5^; while *Fusobacterium* species are associated with colorectal cancer^6^. Moreover, various studies have indicated that an altered microbiome can be associated with disease^7,8^. In HPV-positive women, cervicovaginal bacterial diversity is more complex relative to those who are HPV-negative^9,10^, which indicates that there may be a direct relationship between the cervical microbiome and disease development. In addition to bacteria, viruses have also been shown to be causative agents in disease^11^, with hepatitis B virus (HBV) associated with liver cancer, Epstein–Barr virus (EBV) associated with nasopharyngeal carcinoma, and human papilloma virus (HPV) associated with cervical cancer^12,13^. So far, more and more studies found some virus integration status significantly correlated with disease development status, especially in HPV integration of cervical cancer^14^.

While cervical cancer is one of the most common female cancers worldwide, a detection method that provides an economical, convenient, and accurate clinical screening approach is still required. Current cervical cancer screening methods are usually based on high-risk HPV DNA or RNA genotyping, or on the detection of cytological and/or molecular changes in cervical cells via an immunostaining method, such as a Papanicolaou (Pap) smear^15^. Most of the screening methods just target HPV instead of microbiome. However, several studies have identified a correlation between a microbiome with HPV and cervical intraepithelial neoplasia (CIN) or cervical cancer^16^. One study identified a correlation between the cervical microbiota and CIN stage, with the co-effect of the microbiota and HPV determined to influence CIN risk^17^. *Fusobacteria*, including *Sneathia*, are the species most strongly correlated with HPV infections^17^. Additionally, another study showed that of the vaginal flora, *Lactobacillus*is the dominant species can protect against other pathogens^18^. Other studies have suggested that during an HPV infection, the microbiome balance may be disrupted, with other species, like *Gardnerella vaginalis* and *Chlamydia trachomatis*, meanwhile, *Lactobacillus gasseri* and other anaerobic species becoming more prevalent^9,19,20^. This vaginal microbiome alteration may then affect pathogenesis by affecting immunity or increasing the growth of pathogenic strains^21^. With more and more studies revealing that microbiome alterations can serve as disease diagnostic indicators, with the potential to be used as early screening triage markers for cervical cancer^22^, and aid in the development of analytical methods for detecting the microbiome, such as 16S rRNA sequencing, they have gained interest^23^.

The 16S rRNA gene contains conserved and variable regions that can be used to differentiate microorganisms within the same sample, with the V1 and V9 regions commonly amplified via PCR^24,25^. However, studies utilizing next-generation sequencing technology have predominantly used the V4 or V3V4 regions for identification^26–28^, while other studies have used other regions^29,30^. When determining which region to utilize, some regions provide more information when utilizing short sequencing reads^31^, while others are more informative when a taxonomic approach is employed^32^; therefore, some researchers utilize a full-length 16S rRNA sequence for analysis. With the development of third-generation sequencing technology, it is now possible to completely sequence a full-length gene with a maximum read length up to 2 Mb^33^. In one study, this technology was utilized to sequence the 16S rRNA gene and showed that accurate results can be obtained when compared with Illumina sequencing results^34^. However, this approach is not as portable as employing a Nanopore in the application. In a study evaluating mouse full-length 16S rRNA sequencing obtained using Nanopore or Illumina, with the V3V4 region utilized, the Nanopore sequencing provided better annotation results at the specie level^35^. However, due to the relatively high error rate that is associated with Nanopore sequencing, its application in clinical studies is still very limited.

Nanopore sequencing has been successfully employed in genome assembly, structure variation detection, real-time sequencing of pathogenic microbes, antibiotic resistance profile identification, and the detection and identification of viral or bacterial pathogens in various clinical samples^36–40^; thus, implementing this approach in clinic pathogen and microbiome studies is of interest. Additionally, some studies have identified both viral and bacterial species accurately when utilizing Nanopore amplicon sequencing^41,42^, with NanoAmpli-Seq successfully applied for full-length 16S rRNA gene sequencing and shown to provide a high accuracy^43^. However, while accurate sequencing is an advantage, the library construction process is relatively complex and requires extra data analysis steps and would not be able to provide the quick results that the area expected in a clinical setting.

HPV detection has been clinically proven as imperative for early cervical cancer detection, and the integration status of HPV in patient DNA has also shown to be correlated with cervical carcinogenesis. Studies have shown that the integration sites are distributed throughout the genome, including integration hotspots at 3q28, 17q21, 13q22.1, 8q24.21, and 4q13.3, and are often induced by HPV E1, E6, or E7 proteins^44^. Thus, the ability to collect information pertaining to the HPV infection, HPV integration status, and microbiome status simultaneously would provide valuable clinic information for screening and prevention.

Herein, a novel process was developed to enable the sequencing of an HPV16 E6E7 fragment and a full-length 16S rDNA simultaneously by using multiplex PCR followed by Nanopore sequencing. One clinical sample was examined and shown to be HPV16 positive. The HPV integration sites were identified using a probe capture method, and sequencing was performed using an Illumina sequencer.

## Results

### Nanopore sequencing of multiplex HPV E6E7 and full-length 16S rRNA PCR products

**Figure 1:**
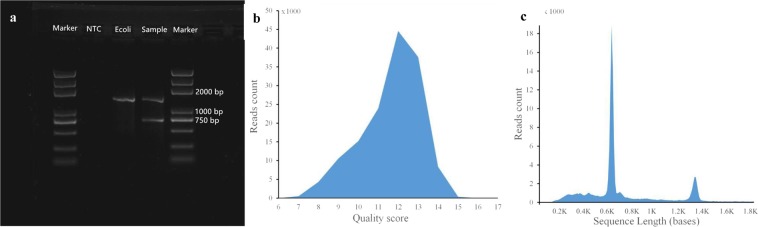
PCR products for Nanopore sequencing and sequencing quality and length distributions. (**a**) Agarose gel of multiplex PCR products, with the full-length gel presented in Supplementary Figure S2. Nanopore sequencing (**b**) Q-score and c. length distribution plots.

Full-length 16S rRNA and HPV16 E6E7 amplicons were obtained as described above, with 750 bp (HPV E6E7) and 1,500 bp (16S rRNA) fragments obtained (Fig. 1a). A Nanopore sequencing library was constructed and sequenced using a Nanopore sequencer. The sequencing generated 189,511 reads from 977 pores. To obtain high-quality reads, the raw data were filtered using the Metrichore 1D base calling program to improve the mean quality score and total read accuracy. A total of 145,605 reads (76.8%; passes 1D reads) were retained with quality scores ranging from 7 to 17, with a mean value of 12.65 (Fig. 1b, Table 1). The read lengths ranged from 88 to 15,068 bp, with a mean read length of 854 bp (Fig. 1c, Table 1).

**Table 1:** Illumina and Nanopore sequencing data statistics.

Platform	Raw reads	Quality filtered reads (≥Q20)	Pass 1D reads	Joined reads	Length (bp)			Sequence quality Scores		
					Min	Mean	Max	Min	Mean	Max
Illumina	1,453,612	1,284,004(88.35%)	/	427,886 (58.88%)	151	249.2	292	19	32.78	35
Nanopore	189,511	/	145,605(76.8%) (120.7M)	/	88	854	15,068	7	12.65	17

### HPV infection and microbiome analysis of Nanopore results

Nanopore sequencing results were analyzed according to the pipeline described in Supplementary Fig. S1 and then evaluated using LAST (http://lastweb.cbrc.jp), with the HPV genome and NCBI 16S ribosomal RNA (Bacteria and Archaea) databases queried following quality score (Q ≥ 7) filtering. The results identified 71,276 reads that matched the HPV16 genome, 48.9% of the passed 1D reads, thus indicating a high amplification efficiency and a high virus load in this sample. Additionally, 17,826 reads matched 16S rRNA sequences, 12.24% of the passed 1D reads. The remaining unmatched reads totaled 56,503 (Supplementary Table S1).

To analyze the microbiome data more accurately, QIIME (Quantitative Insights into Microbial Ecology) was used to assign taxonomy annotations at all classification levels from phylum to genus (Supplementary Tables S2 and S3). At the phylum level, four phyla were identified, including Firmicutes, Proteobacteria, Bacteroidetes, and Actinobacteria, with Firmicutes being the most abundant (98.9%). Seven classes were identified, including Bacilli, Clostridia, Bacteroidia, Actinobacteria, Alphaproteobacteria, Betaproteobacteria, and Gammaproteobacteria, with Bacilli being the most abundant (96.6%). Additionally, 8 orders and 15 families were identified, with Lactobacillales and Enterococcaceae being the two most abundant families among those tested. In total, 21 genera were identified, with *Enterococcus* being the most abundant (94.1%). Bacteria associated with bacterial vaginosis (BV)^45^ were also examined, and six genera were identified, including *Bacteroides*, *Prevotella*, *Enterococcus*, *Streptococcus*, *Staphylococcus*, and *Peptostreptococcus* (Supplementary Table S4).

### Taxonomic annotation comparison between the 16S rRNA Illumina V4 results and the Nanopore 16S full-length results

To evaluate the accuracy of the Nanopore results, the 16S rRNA V4 region of the same sample was amplified by PCR and sequenced using an Illumina next-generation sequencer. In total, 1,453,612 raw reads (219.5 M raw bases) and 1,453,363 clean reads (217.3 Mb) were obtained when using the Illumina sequencing platform (Table 1), with 1,284,004 (88.35%) reads retained after quality filtering. A total of 427,886 (58.88%) sequencing reads were then joined, with lengths between 151 bp and 292 bp and an average length of 249 bp. The quality score range was from 19 to 35, with an average of 32. Approximately 4% (27,123) of the sequencing reads were retained for further microbial community analysis (Table 1).

**Figure 2:**
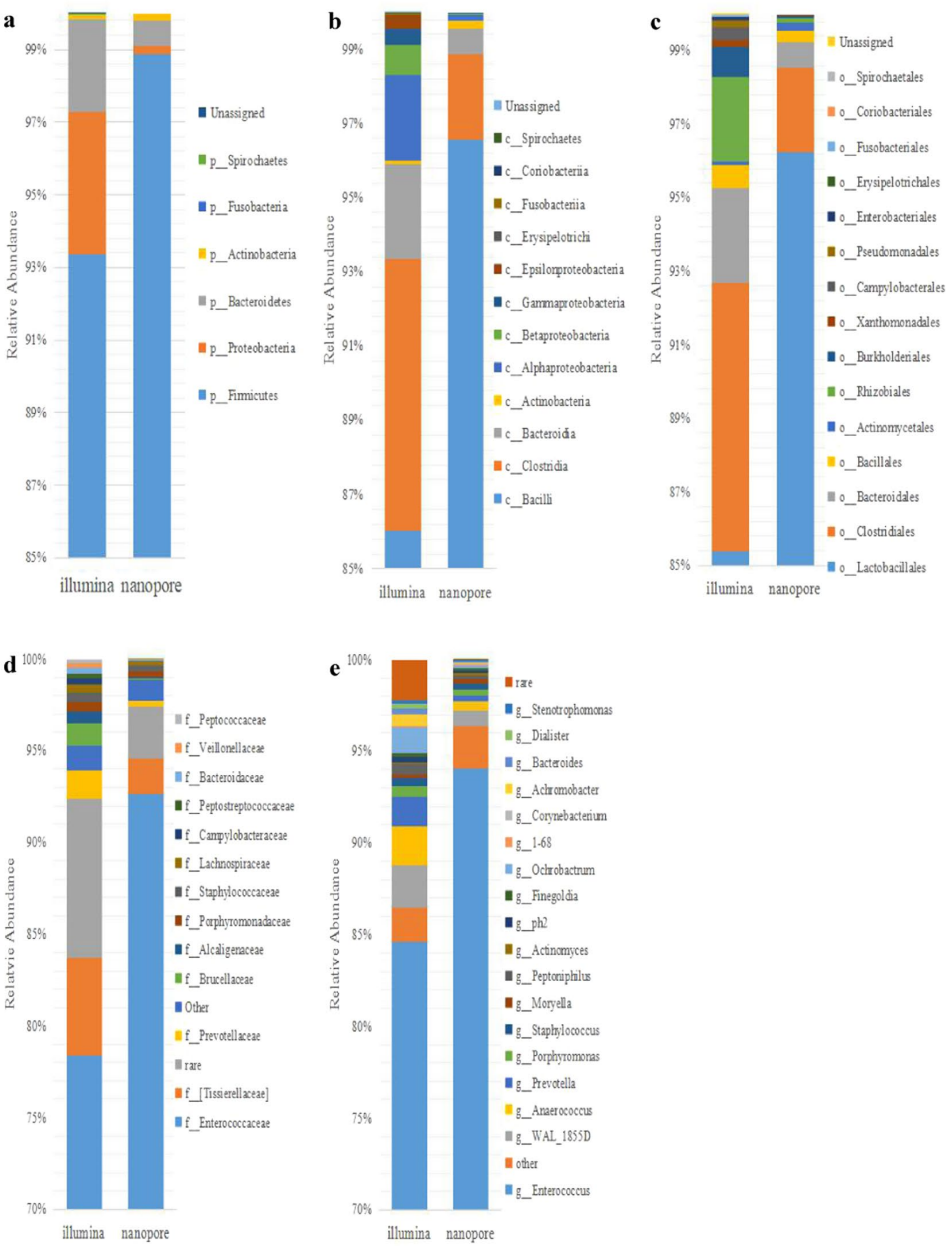
Comparison between the taxonomic profiles obtained from two different sequencing platforms at different taxonomic levels (phylum to genus). (**a**) phylum level, (**b**) class level, (**c**) order level, (**d**). family level, and (**e**). genus level.

The paired passing filter reads were classified using QIIME and were grouped into 7 phyla, 13 classes, 16 orders, 32 families, 50 genera, and 7 species. The annotation results from the Illumina platform were then compared with the Nanopore results at the phylum, class, order, and genus levels (Fig. 2, Supplementary Tables S2 and S3). When examining the bacteria, both platforms identified 4 phyla, 7 classes, 10 orders, 15 families, and 20 genera (Supplementary Tables S2 and S3). Several conclusions can be drawn from the results: (1) both of the platforms identified p_Firmicutes, c_Bacilli, o_Lactobacillales, f_Enterococcaceae, and g_Enterococcusas being a significantly dominant population, with 85% and 94% relative abundances in the Illumina and Nanopore results, respectively. (2) The Illumina results have more unique taxonomic units than the Nanopore results, with the Nanopore results having only one unique genus (*Vagococcus*, 0.035%), while the Illumina results had multiple at the phylum (3/7), order (6/16), class (6/13), family (18/33), and genus (31/51) levels. These findings suggest that the Illumina platform is more sensitive and can detect more bacterial categories when compared with Nanopore. The difference could also be attributed to Illumina generating larger datasets with more sequencing depth or differences in the QIIME pipeline for short and long sequence reads. (3) While Illumina identified more unique taxonomic units, the two platforms generated an overlapping bacteria list for each level, with orders having an 80% overlap and belonging to the top 10 orders, while other units were all associated with the highest units (Supplementary Table S2). Based on the calculated high abundance taxonomic unit results, there is only one unique Illumina units in the top 5 phyla, 3 in the top 10 classes, 2 in the top 10 orders, and none in the top 15 families or top 20 genera. These findings indicate that most of the unique units identified by using the Illumina V4 method are at a low abundance and may be attributed to an experimental or analytical bias. (4) Without performing any additional analytics, the identified taxonomic unit results when comparing the Nanopore and Illumina results show a strong concordance among the most abundant bacteria at each level (Supplementary Table S3). When comparing the two platforms, the Spearman’s rank correlation results gave a 0.9265616 at the phylum level, 0.8796751 at the class level, 0.8409996 at the order level, 0.7095144 at the family level, and 0.6583039 at the genus level. In conclusion, the Nanopore platform detected the dominant *Enterococcus* genus and 20 other genera that overlap with the Illumina V4 results. Furthermore, when examining the 16S rRNA, they were 100% consistent at the genus level, which strongly suggests that the Nanopore platform can provide acceptable results by sequencing a full-length 16S rRNA. (5) BV-related bacteria within the Illumina results were examined, and 10 genera were identified, with *Bacteroides*, *Prevotella*, *Enterococcus*, *Streptococcus*, *Staphylococcus*, and *Peptostreptococcus* also identified by the Nanopore platform (Supplementary Table S4).

### Specie annotation result comparisons when using different analytical methods and databases

**Figure 3:**
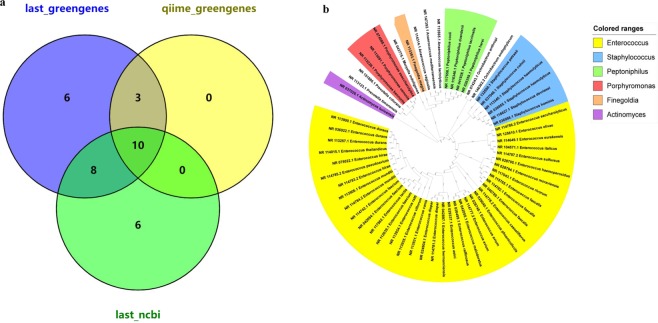
Nanopore 16S rRNA results when overlapping different databases. (**a**) A venn diagram displaying the findings for different databases at the genus level. (**b**) A phylogenetic tree of species annotated using the LAST_NCBI method.

Nanopore results were further annotated at the specie level using QIIME and LAST, with the GreenGenes database, and the methods were named QIIME_GreenGenes and LAST_GreenGenes. The results annotated 5,574 reads to 23 genera (reads ≥ 2), with only 3 species clearly annotated using the QIIME_GreenGenes method, while the reaming 20 genera could not be annotated using this method (Fig. 3a, Supplementary Tables S5 and S6). The LAST_GreenGenes method identified 53 genera (reads ≥ 2) and annotated 19 species, with only 6 species of the overlapped genera identified. Since most of the genera were without specific specie annotations, the LAST_NCBI database was utilized to attempt to obtain better annotation results. By using this method, all 127 genera (reads ≥ 2) were annotated with at least one specific species. Overall, 197 specific species were found, including 70 species belonging to the 10 overlapped genera (Table 2, Supplementary Tables S5 and S6). When comparing all three of the analytical methods, only two genera, *Enterococcus* and *Staphylococcus*, had specie annotations in all three methods. When using the LAST_NCBI method, 7 of the 10 overlapped genera were identified in multiple species, especially *Enterococcus*, with over 90% of the identified reads annotated to 33 specific species. Another genus, *Peptoniphilus*, was classified to eight specific species (Supplementary Tables S5 and S6). Overall, these comparison results clearly show that a significant improvement in specie annotations can be obtained when using the LAST_NCBI analysis method.

**Table 2:** Summary of the species that were identified when utilizing the 10 genera that were commonly identified using three different analytical methods.

Overlapped Genus	Number of Identified Species		
	QIIME_greengenes	Last_greengenes	Last_NCBI
g__*Actinomyces*	0	0	1
g__*Anaerococcus*	0	0	7
g__*Enterococcus*	1	2	33
g__*Finegoldia*	0	0	1
g__*Moryella*	1	0	1
g__*Ochrobactrum*	0	0	3
g__*Peptoniphilus*	0	0	8
g__*Porphyromonas*	0	0	4
g__*Prevotella*	0	0	5
g__Staphylococcus	1	4	7
Total	3	6	70

These bacterial strains (reads ≥ 5) were annotated using the LAST_NCBI method and were used to construct a phylogenetic tree (Fig. 3b). The phylogenetic tree showed that the strains classified into five separate groups, with *Actinomycesturicensis* in a separate outside branch. *Enterococcus* was the most abundant genus, with 27 different species (reads ≥ 5) and 37 strains, and comprised the first group. The second and third groups included *Staphylococcus* (6 species) and *Ochrobactrum* (2 species; reads ≥ 5). *Anaerococcus* (n = 3), *Finegoldia* (n = 1), *Moryella* (n = 1) and *Peptoniphilus* (n = 4) strains all comprised the fourth group. The fifth group included two genera, *Porphyromonas* (n = 3) and *Prevotella* (n = 2).

### Rapid clinical sample pathogen detection via Nanopore MinION

To evaluate the minimum sequencing time required for both HPV16 and full microbiota detection, different percentages (5%, 10%, 15%, 20%, 25%, or 30%) of the total sequencing reads were extracted four times, and the microbiota composition was compared with the full sequencing results (Table 3 and Supplementary Tables S7 and S8). The results showed that when extracting as few as 4,459 reads from the total sequenced reads, the Nanopore platform can detect the top 10 abundant species that were identified when utilizing all of the sequencing results. Statistical and correlation coefficient analyses showed that there is no significant difference when using a subset of the reads or the total number of reads in terms of identifying bacterial species. These findings indicate that using this Nanopore amplicon library sequencing method can enable both HPV and microbiota species to be identified in as short as about 15 min when using this sequencing process. Overall, from the point of DNA extraction through sequencing, with sequencing times varying from 10 min to several hours, the process takes an average of 6 h. However, while the combination of multiplex PCR and Nanopore sequencing to detect HPV virus and other potential microbial pathogens in clinical samples looks promising, further examination into the applicability of this rapid detection method is required.

**Table 3:** Summary results from different extracted Nanopore sequencing reads.

Ratio	Estimated sequencing time (min)	Extracted reads (avg.)	16S (avg.)	HPV (avg.)	TOP10 bacteria reads (avg.)	TOP10 bacteria numbers (avg.) (reads > 4)	Correlation coefficiency with all	*P*- value
5%	15	4,459	919	3,540	859	7	0.999	0.109
10%	30	8,851	1,768	7,083	1,661	9	0.999	0.109
15%	45	13,332	2,668	10,664	2,503	10	0.999	0.109
20%	60	17,801	3,606	14,195	3,384	10	0.999	0.109
25%	75	22,295	4,432	17,863	4,141	10	0.999	0.109
30%	90	26,780	5,362	21,418	5,035	10	0.999	0.109
all	300	89,102	17,826	71,276	16,672	10	1	/

### HPV integration site detection and verification

**Figure 4:**
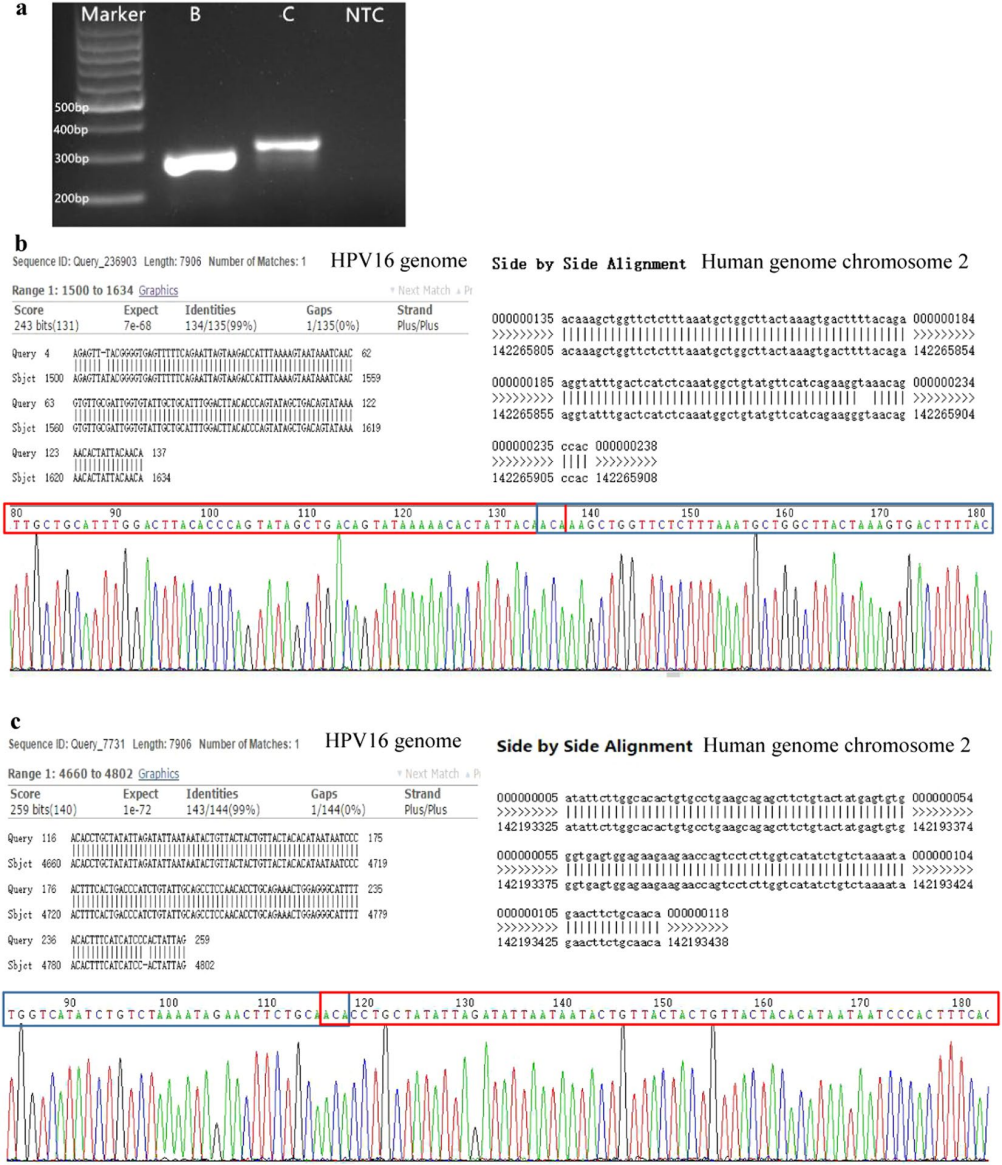
Two Sanger verified HPV-*LRP1B* integration sites. (**a**) PCR products for the B and C integration sites, with the full-length gel presented in Supplementary Figure S3. (**b**,**c**). HPV and human *LRP1B* gene Blast results and Sanger sequencing results for the verified B and C integration sites.

HPV integration sequences were further enriched and sequenced by using probe capture and Illumina sequencing. HPVDetector was used to analyze the integration sites and the results identified a number of integrations, with HPV16 E1 and L2 (NC_001526.4) significantly enriched in *LRP1B* on chromosome 2 within the human genome (GRCh37/hg19). The integration site distributions are shown in Supplementary Tables S9 and S10, and the integration sites located within 200 bp are recognized as one. In the *LRP1B* gene, 4 sites were found to be integrated with HPV16 E1 or L2, with at least 10 sites identified by HPVDetector (Table 4). The location of HPV and *LRP1B* was calculated based on the average values of the merged locations. Overall, a total of 461 potential HPV integration sites were discovered in 22 different genes (Supplementary Tables S9 and S10). These identified high integration sites were then validated using Sanger sequencing, and a total of two unique HPV16 integration sites in the *LRP1B* gene were verified (Fig. 4). Therefore, these findings confirm that this patient is not only HPV16 infected but also HPV16 integrated.

**Table 4:** *LRP1B* integration sites identified using HPVDetector (n > 10).

HPVtype	HPV integration location	Chromosome	Chr. integration location	HPV gene	Human gene	Number of identified
HPV16	4,687	2	142,193,255	L2	*LRP1B*	224
HPV16	4,721	2	142,272,444	L2	*LRP1B*	20
HPV16	908	2	142,175,473	E1	*LRP1B*	10
HPV16	1,458	2	142,265,838	E1	*LRP1B*	170

## Discussion

The human microbiota is a complex ecosystem of diverse microorganisms consisting of bacteria, fungi, and viruses that predominantly reside in epidermal and mucosal regions across the body^23^. To investigate the role of microbiota in human health, including microbiotahost interactions and microbiota associations with diseases, especially cancers^46,47^, several methods have been employed for determining the microbial community composition from clinical samples^48^. Conventionally, microbial diversity analyses have focused on the V4 hypervariable region of the 16S rRNA gene and have utilized the Illumina platform^49^. However, the short sequencing reads obtained when using this platform often limit the microbial composition analysis at the species level due to a high similarity between the 16S rRNA amplicon sequences^35^. To overcome this limitation, obtaining a full-length 16S rRNA gene sequence using the Nanopore MinION sequencing platform has been explored^29,50,51^. In this study, a new strategy enabling the simultaneous analysis of an HPV infection and microbiomes using Nanopore sequencing was explored and shown to be effective.

Other studies have attempted to perform a more rapid Nanopore data analysis, with one study establishing a relatively simple workflow for rapid bacterial identification via MinION™sequencing using a mock bacteria community instead of using a real environmental or clinical sample^52^. Furthermore, another study established an amplicon sequencing (AmpSeq) workflow for predicting Newcastle disease viral virulence and genotype and was able to correctly identify NDV genotypes in all serial dilutions with an accuracy of 98.37%~100% after only 7 min of sequencing^53^. Additionally, another study developed a portable system for analyzing 16S rRNA using a Nanopore sequencer and was able to detect 20 bacteria in a mock sample after 5 min of sequencing, with the results being consistent with those obtained after 4 h of sequencing^54^. Herein, data analysis and sequencing were not performed simultaneously, but the above procedure was mimicked by partially extracting the data and analyzing it separately. The results presented herein show that even a small portion of the Nanopore data can identify high abundance bacteria accurately when compared with the results analyzed from all of the Nanopore sequencing reads. Future studies should focus on establishing a bioinformatics procedure that can combine the Nanopore sequencing and the detection of clinic pathogens in a timely fashion.

Some studies have indicated that third-generation sequencing, like Pacbio, has advantages in bacteria species identification when compared with 16S rRNA sequence using Illumina^34,55,56^. Herein, a similar conclusion was not drawn, possibly due to this study utilizing the GreenGenes database for taxonomy annotations. This database did not have specie annotations for some of the identified reads, especially for the genus *Enterococcus* that comprised over 80% of both the Illumina and Nanopore sequencing results. To improve the annotations on the species level, the NCBI database was also employed and 15,221 bacterial species were identified, compared to the 4,309 bacterial species that were identified with the GreenGenes database. However, the NCBI database is not compatible with the QIIME software that was needed to compare the two platforms. Thus, the development of a database with more up to date species annotations would improve future studies and enable a more thorough evaluation of the advantages of third-generation sequencing of a full-length 16S rRNA^56^.

The Nanopore MinION sequencer has the advantage of being portable and cost effective^55,57^. While the error rate of this platform is still under debate, it shows clinical applicability for use as a rapid detection system for viral or bacterial detection^50,58–60^. Various studies have shown that utilizing certain data correction methods can significantly minimize the error rate. For example, CANU^61^ and MECAT^62^ can be used to do self-correction with Nanopore sequencing data or hybrid Illumina/Nanopore datasets, with this improved accuracy potentially broadening the applicability of this technology. Furthermore, some studies have even used this platform to accurately sequence antibiotic resistance sites^63^. Herein, only LAST was utilized to analyze and annotate the Nanopore sequencing. While the exact error rate of the sequencing data was not determined, the results showed a high degree of comparability with the Illumina results. One major reason could be that the most abundant microbe was present in several genera, thus providing enough coverage of the genus to annotate. In addition to the most abundant genera, the other genera with a relative abundance of about 0.03% could still be detected. These findings indicate that this method is feasible for detecting low abundance bacteria in clinic samples. However, further studies need to establish the lowest limit of detection.

The liquid-based cytology sample used in this study represents a huge population of clinical resources that are routinely collected yearly. The main screening methods for cervical cancer are Papanicolaou (pap) smear, cervical liquid-based cytology, cervicography, cervical biopsy or HPV testing^64–66^. The liquid-based cytology (LBC) is widely used in the detection of gynecological specimens, vaginal or uterine, for the screening or diagnosis of cervical epithelial lesions^67^. Recent reports have increasingly focused on utilizing LBC in combination with HPV-DNA detection when screening for cervical cancer to improve accuracy and sensitivity in pathogen detection^19,21,67,68^. Studies examining correlations between the cervical BV-related microbiome, HPV screening and HPV infection, and cervical microbiome alterations have shown that there is a certain correlation between an HPV infection and the occurrence of cervicitis, cervical lesions, and cervical cancer^69–71^, while the vaginal microbiome plays a functional role in the persistence or regression of HPV infections and the occurrence of cervical cancer^8,72^. Lee *et al*. showed that *Fusobacteria*, including *Sneathia* spp., can serve as a possible HPV microbiological marker^73^. Furthermore, another study reported that the vaginal microbiome in high-grade (HSIL) samples is characterized by higher levels of *Sneathia sanguinegens* (*P* < 0.01), *Anaerococcus tetradius* (*P* < 0.05), and *Peptostreptococcus anaerobius* (*P* < 0.05), while lower levels are characterized by *Lactobacillus jensenii* (*P* < 0.01) when compared to intra-epithelial lesions (LSIL)^8^. Therefore, collecting all of the information for every woman as early as possible so the microbiota associated with healthy and early disease states can be better characterized is essential to aid in disease prevention or treatment.

In one study, a similar type of sample was used, and the microbiome was identified using a proteomic approach, but only limited information could be detected^74^. Herein, a method based on molecular nucleotide information was utilized to define the microbiome information by successfully analyzing a liquid-based cytology sample and identifying an HPV infection and bacterial content. Although only one sample was used in this study, the findings support its future applicability of this method to define HPV and its types and infections. In this study, only one primer was designed, HPV16 E6E7, but there are tens of high-risk HPV types that exist. Of these, some types are prevalent in human samples, including cervical samples, head and neck samples, and more than ten kinds of human organ-related samples. Furthermore, full-length 16S rRNA sequencing can promptly provide a patient pathogen profile to aid in performing a clinic diagnose, including applications in bacteria discovery in meningitis^75^, liver abscess^40^, and empyema^54^. Thus, this workflow developed to detect HPV and the microbiome can be expanded to study other clinic samples and can aid in clinic diagnostic applications outside of cervical cancer.

In cervical carcinogenesis, HPV integrates into the host genome following a break in the E2 gene, which has been described as the main repressor of the expression of the E6 and E7 oncogenes and is a key genetic event in cervical carcinogenesis^9,16,17,76^. The level of HPV integration was reported to be positively correlated with cervical intraepithelial neoplasia (CIN) grades and has even been proposed as a marker for disease progression^77,78^. Moreover, another study found that HPV integration in *LRP1B* decreases its protein expression^70^. HPV integration in *LRP1B* has also been found in oropharyngeal squamous cell samples^79^. This study identified and verified two major HPV16 integrations (E1 and L2) in the human *LRP1B* gene. While many other integration sits were sequenced by the next-generation sequence, Sanger sequencing can only detect high abundance sequences due to the limitation of the technique. Thus, the other identified integration sites will have to be verified using other more sensitive molecular detection methods that are able to verify low abundance sequences in the future. Nevertheless, these results show correlations with previous studies. This study showed that this patient was not only infected with HPV16, but the virus was also integrated, thus indicating a potentially carcinogenic attribute to cervical cancer which requires further attention. Overall, identifying HPV integration breakpoints in the human genome and elucidating the mechanisms of integration can enable a better understanding of HPVinduced cervical carcinogenesis. Furthermore, it is also beneficial to discover novel and more specific biomarkers for diagnosis and treatment.

At present, this study detected HPV-DNA within a LBC sample following multiplex PCR and Illumina and Nanopore sequencing. Moreover, both the HPV and microbiota were characterized by using multiplex PCR technology using MinION. This not only shows the relationship between HPV integration and cervical cancer progression but also reveals a correlation between the bacterial community and the HPV integration status, which can aid in the early diagnosis and treatment of cervical cancer.

## Conclusions

Herein, a novel approach was developed to enable the simultaneously detection of HPV and bacteria in a human LBC sample using Nanopore sequencing. This approach expands the pathogen detection potential of the Nanopore platform and offers rapid results that are desirable in a clinical setting. This study also showed that Nanopore sequencing of full-length 16S rRNA can provide comparable microbiota results to those obtained when utilizing the Illumina V4 sequencing method, with the top most abundant genera consistent between platforms. Additionally, four HPV16 L2 or E1 integration sites within the *LRP1B* gene were identified, with two of them verified using Sanger sequencing. A deep analysis was performed to examine HPV infection and microbiome features associated with the examined patient sample to establish a broad potential application for HPV and microbiome analysis in a clinical setting. This approach can potentially be utilized as a diagnostic tool or can be potentially be utilized in other research and application areas.

## Material and Methods

### Sample information

Exfoliated cervical epithelial cell samples were collected and diagnosed as negative for intraepithelial lesions or malignancy (NILM) using the LBC. The sample used in this study was collected at the Sanmenxia Central Hospital, Henan Province, China, and the patient was 66 years old. The sample was collected for an HPV screening test, and the residual sample was used for this study. The sample was found to be HPV16 positive using a fluorescent HPV Genotyping kit (Bioperfectus Technologies, Jiangsu, China). This study was approved by the Medical Ethics Committee of Sanmenxia Central Hospital, Henan, China (Consent Number PROT No.36 [2018]). All experiments were performed in accordance with relevant guidelines and regulations and informed consent was obtained from the patient.

### Genomic DNA extraction

Genomic DNA (gDNA) was extracted from exfoliated cervical epithelial cells using a TIANamp Micro DNA Kit (Tiangen, Beijing, China) according to the manufacture’s protocols. Briefly, samples were collected and stored in 1.5 ml sterilized tubes at 4 °C. A cell pellet was then formed following centrifugation at 5,000 rpm for 5 min and the DNA was extracted. The double-stranded (ds) DNA concentration was quantified using a Qubit dsDNA HS Assay Kit and Nanodrop 2000 (Thermo Fisher Scientific, Inc., Waltham, MA, USA).

### Amplification of 16S V4 and Illumina sequencing library construction

The isolated gDNA was used as a template to amplify the V4 hypervariable region of the 16S rRNA gene^80^. PCR was performed in a total volume of 20 µL and contained 10 µL KAPA HiFi HotStart Ready Mix (KAPA Biosystems, Wilmington, MA, USA), 0.5 µL each primer (10 nM), and 20 ng gDNA. Reactions were initially heated to 94 °C for 3 min, followed by 5 cycles at 94 °C for 30 s, 45 °C for 20 s, and 65 °C for 30 s; 10 cycles at 94 °C for 30 s, 50 °C for 30 s, and 72 °C for 30 s; and 15 cycles at 94 °C for 30 s, 55 °C for 30 s, and 72 °C for 30 s. Reactions were completed at 72 °C for 5 min.

Amplified PCR products were purified using 1.6× Agencourt AMPure XP beads (BeckmanCoulter Genomics, Brea, CA, USA) and then verified using 2% agarose gel electrophoresis. The purified DNA was then used to construct Illumina libraries using the NEBNext^®^UltraII^TM^ DNA Library Prep Kit for Illumina^®^ (E7370L; New England Biolabs, Ipswich, MA, USA) and NEBNext^®^ Multiplex Oligos for Illumina^®^ (E6609L; NEB) according to the manufacturer’s instructions. The generated library was quantified using a Qubit dsDNA HS Assay Kit (Thermo Fisher Scientific) with a Qubit 3.0 fluorometer (Invitrogen) and qualified using an Agilent 2100 TapeStation (Agilent Technologies, Santa Clara, CA, USA). The libraries were paired-end (2 × 150 bp) sequenced using an Illumina NextSeq Mid Output platform with a NextSeq. 500/550 Mid Output v2 kit (300 cycles) in rapid run mode according to standard Illumina sequencing protocols.

### HPV and 16S rRNA amplification

The gDNA obtained from the LBC was used as a template and the full-length 16S gene was amplified using specific primers (S-D-bact-0008-c-S20 and S-D-bact-1391-a-A-17)^26^, while simultaneously amplifying the HPV16 genome using HPV16 E6E7 specific primers (NC_001526.2; Supplementary Table S11). The PCR reaction was carried out in a total volume of 25 µL containing 12.5 µL 2x GC buffer I (TaKaRa, Shiga, Japan), 2 µL dNTPs (2.5 µM), 0.5 µL each of forward and reverse primer (10 µM), 0.5 µL LA Taq® with GC Buffer (TaKaRa; 125 U), 2 µL template DNA (20 ng), and 1.0 µL nuclease-free water (not DEPC-treated). The amplification conditions were as follows: 4 min at 94 °C; then 30 cycles at 94 °C for 30 s, 50 °C for 40 s for annealing, and 72 °C for 90 s; and a final 72 °C for 15 min. PCR products were verified via gel electrophoresis and cleaned-up with AMPure XP beads (Beckman Coulter, Miami, FL, USA). Amplicons were quantified using a Qubit 3.0 fluorometer (Life Technologies, Carlsbad, CA, USA), and 1000 ng of the purified amplicon was used to generate a MinION library.

### Amplicon DNA library preparation and ONT sequencing

The amplicons were prepped using a Ligation Sequencing kit (SQK-LSK108; Oxford Nanopore Technologies) with Native Barcoding Expansion (EXP-NBD103; ONT), according to the manufacturer’s protocol for 1D Native barcoding gDNA. The prepared library (12 µL at ~158 ng) was then combined with 35 µL of running buffer containing Fuel Mix (RBF) and 25.5 µL Library Loading beads (LLB, ONT) and loaded into a R9.4 flowcell (ONT) via the SpotON port according to the manufacturer’s instructions.

### Nanopore sequencing data analysis

The Nanopore sequencing results were base called using the EPI2ME interface (v. 2.59.1896509). For passed 1D reads, quality scores (Q-score ≥ 7) and length distributions were evaluated using FastQC. Obtained fastq files were then converted to fasta files using the FASTX-toolkit ((http://hannonlab.cshl.edu/fastx_toolkit/) and the sequences were aligned with the HPV genome database using LAST, with default parameters. Non-HPV reads, HPV reads and HPV type information was obtained. The non-HPV reads were filtered based on length, with only sequences >1.2 Kb or <1.6 Kb retained. For the retained non-HPV reads, taxonomies were annotated using the Greengenes database in QIIME^81^, with a ≥90% similarity level required. To further compare the results and obtain more through specie annotation results, LAST aligner^82^ was used in conjunction with a subset of the Greengenes database^83,84^ and the NCBI database. The obtained data was then evaluated by constructing a phylogenetic tree based on full-length 16S rRNA sequences from species (reads ≥ 5) identified by using the NCBI database and based on 10 genera that were commonly identified using three different analysis methods. The phylogenetic tree was visualized using MEGA X (https://www.megasoftware.net).

### Illumina sequencing data analysis

To analyze the Illumina sequencing quality, FastQC version 0.11.8 (http://www.bioinformatics.babraham.ac.uk/projects/fastqc/) was utilized, and then the sequence reads were merged using FLASH-1.2.11 (Fast Length Adjustment of Short Reads; http://ccb.jhu.edu/software/FLASH/)^85^ with a minimum overlap of 10. QIIME was used to filter and cluster combined pairs, with a quality score <Q20 and read length <265 bp. These high-quality reads were then clustered using pick_otus.py in QIIME, with the UCLUST greedy algorithm utilized at a 97% similarity threshold. Taxonomies were defined using the GreenGenes database in QIIME at a 90% similarity threshold.

### Comparison of Illumina and Nanopore sequencing data

The sequencing data were compared using the R statistical package version 3.6.0 (https://www.r-project.org), and Spearman’s rank correlation tests were performed using the vegan package. Heatmaps and Venn diagrams were generated using the ggplot2 package within R.

### HPV integration site sequencing and analysis

Integrated HPV sequences were enriched using HPV probes (Integrated DNA Technology, IDT) and an Illumina sequencing library was constructed using a NEBNext^®^UltraII^TM^ DNA Library Prep kit for Illumina^®^ (E7370L) with NEBNext^®^ Multiplex Oligos for Illumina^®^ (E6609L) as recommended by the manufacturer. HPVDetector was used for the integration site analysis as previously described^86^.

### Integration site verification using Sanger sequencing

Primers were designed using Primer Premier 5.0, with two fusion sites with a high detection frequency targeted to generate an amplicon fragment size of 200 ~ 300 bp. The forward primers were designed based on HPV sequences, and the reverse primers were designed based on human genome sequences. B primers and C primers are displayed in Supplementary Table S11.

Sequences were amplified using Phoenix™ Hot Start *Taq* DNA Polymerase (Enzymatics) in a PCR reaction mix containing 4 µL 5X Phoenix Hot Start Taq Reaction Buffer, 2 µL dNTPs (2.5 µM), 0.5 µL each of forward and reverse primer (10 µM), 0.2 µL Phoenix™ Hot Start Taq DNA Polymerase (500 U), 1 µL template DNA (10 ng), and 12.3 µL Nuclease-Free Water (not DEPC-treated), for a total volume of 20 µL. The PCR amplification conditions were as follows: 5 min at 95 °C; 35 cycles at 94 °C for 30 s, 60 °C for 60 s for annealing, and 72 °C for 60 s; and a final 72 °C for 1 min. PCR products were visualized via agarose gel electrophoresis and cleaned-up using AMPure XP beads (Beckman Coulter, Miami, FL, USA).

## Supplementary information

Supplementary Figure S1-S3

Supplementary Table S1-S11

## Data Availability

The datasets and analyses generated during this study are available in the Genbank database repository (accession: PRJNA545852; https://www.ncbi.nlm.nih.gov/gen -bank/).
