# Supplementary material for: Simultaneous detection and comprehensive analysis of HPV and microbiome status of a cervical liquid-based cytology sample using Nanopore MinION sequencing
Source: Sci Rep. 2019 Dec 18;9:19337. doi: 10.1038/s41598-019-55843-y (PMC6920169; doi:10.1038/s41598-019-55843-y)

Simultaneous detection and comprehensive analysis of HPV and microbiome status of a cervical liquid-based cytology sample using Nanopore MinION sequencing

Lili Quan, Ruyi Dong, Wenjuan Yang, Lanyou Chen, Jidong Lang, Jia Liu, Yu Song, Shuiqing Ma, Jialiang Yang, Weiwei Wang, Bo Meng, Geng Tian.

Supplementary Figure S1. The analytical pipeline of Nanopore sequencing data.

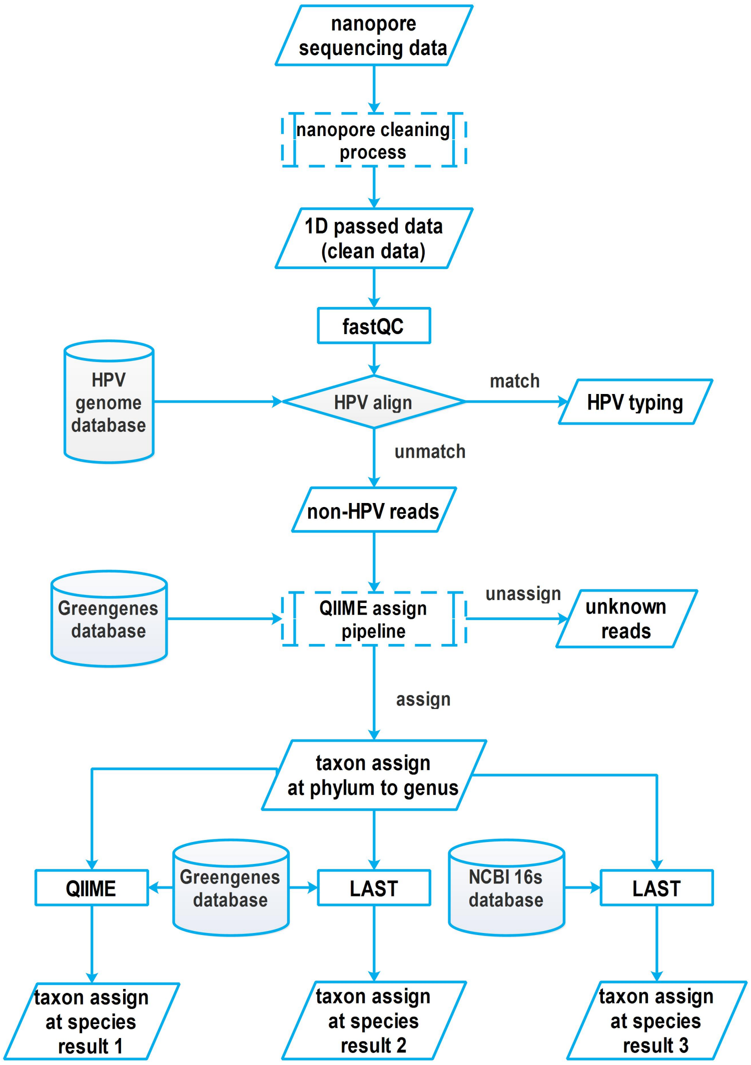

Supplementary Figure S2. The full-length agarose gel of multiplex PCR product.

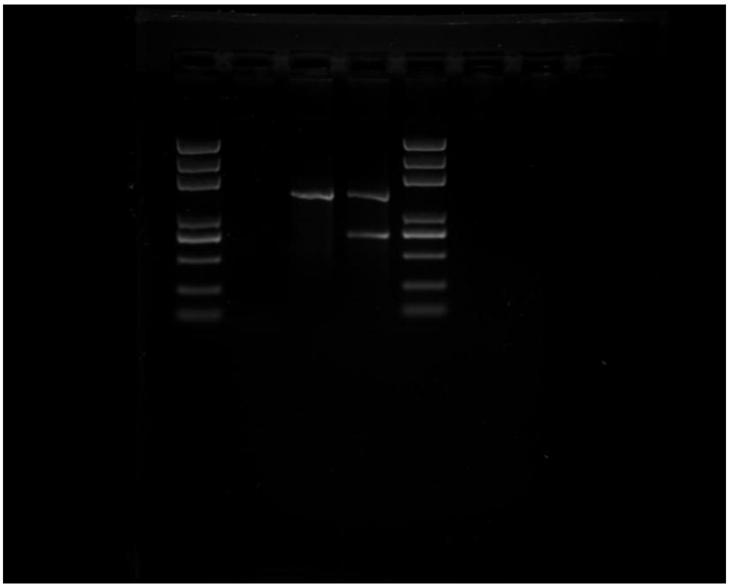

Supplementary Figure S3. The full-length gel of PCR products for integration B and C.

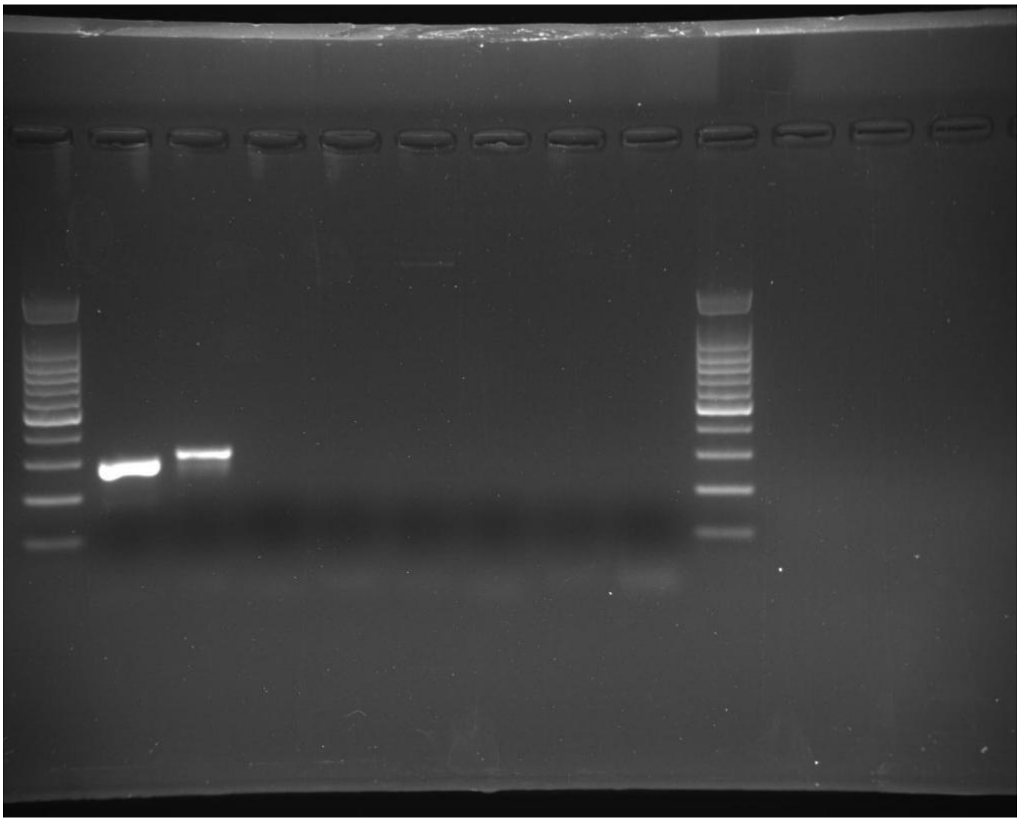

Supplement: Supplementary file 1 — Supplementary Figure S1-S3 [file 41598_2019_55843_MOESM1_ESM.pdf]
